# Who owns your data? Part II ownership of your scholarly article

**DOI:** 10.1002/acm2.12393

**Published:** 2018-07-01

**Authors:** Michael D Mills

You are putting the final touches on your latest article and it is time to consider a venue for publication. It is also time to ask yourself some questions. What is the urgency of publication? Am I going to use this article for educational purposes? Do I have a need to post the article in a public forum?

The AAPM's approach to academic dissemination is to provide complementary publishing options, based on the article's content and the preference of the authors. The open access model of the JACMP is not in conflict with those of traditional publications, such as our companion publication, *Medical Physics*. Even so, traditional publishers are adjusting their publishing models to keep abreast with the changing landscape of publishing trends. These include the following:

- Many traditional publications have begun to offer an open access option. By paying an additional fee, any article may be offered to the public without cost.
- Articles may be offered in public archives prior to being submitted to traditional journals and peer review. An example of this is the arXiv repository: https://arxiv.org/
- Some traditional journals are maintaining a subscription model and a paywall but abandoning print as a mode of delivery. This is because print is too costly to maintain unless there is robust print advertising to cover the cost.
- Print journalism is expected to expire in about 10 years. Could academic journals be close behind? http://www.dailymail.co.uk/news/article-5385169/New-York-Times-stop-printing-10-years-says-CEO.html

If you decide to publish in a traditional print journal, you are expected and required to sign a copyright transfer agreement. For publication in *Medical Physics*, the copyright is transferred to the AAPM and the society becomes the owner of your article. Distribution of your article is subject to the restrictions enumerated in the publication agreement between the AAPM and Wiley. The copyright transfer agreement is appended for your interest (Fig. 1). If you believe that you are reaching your intended audience by publishing in a traditional print journal, there is no disadvantage to relinquishing your copyright.

**Figure 1:**
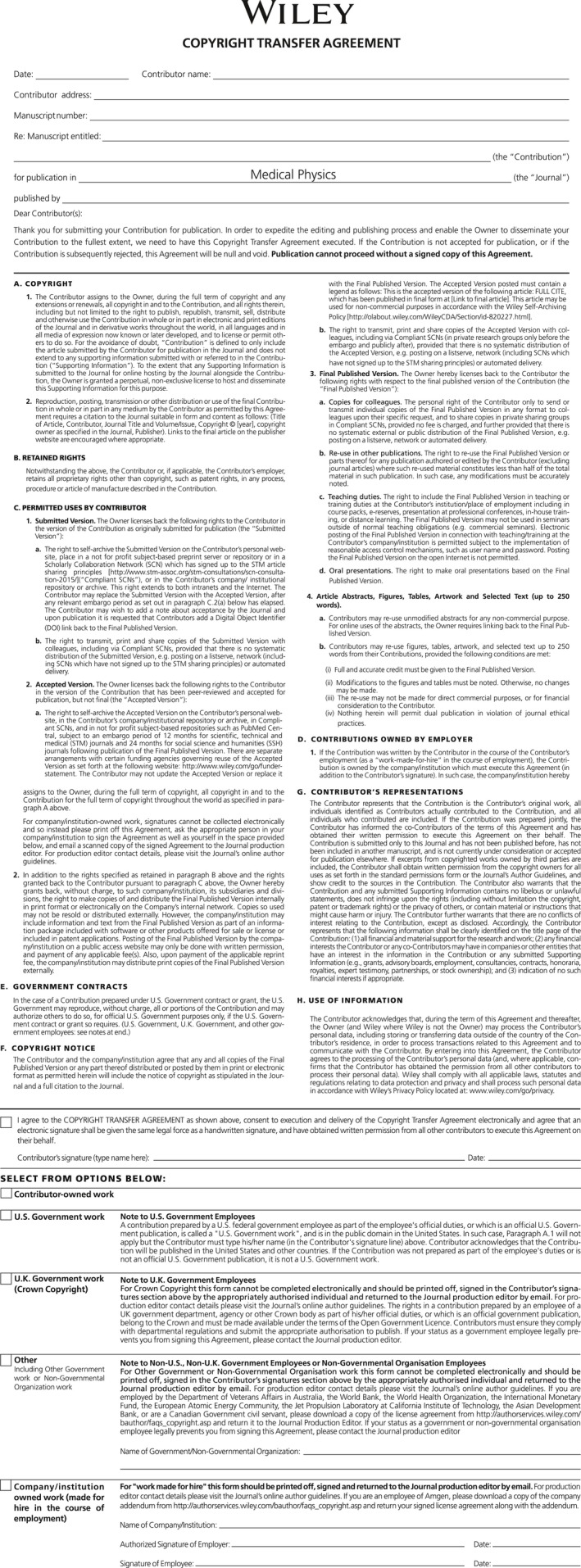
The AAPM copyright transfer agreement for *Medical Physics*.

What factors are important to consider when you are deciding whether to publish open access? Some that come to mind include the cost and speed to publication as well as the visibility of your article and the benefit that you retain the copyright. For the JACMP, the article publication charge of $500 is among the lowest in the industry. The time from receipt at Wiley to Early View publication is typically between 30 and 40 days. Also, if you select an open access journal for your article, more people are likely to access and read it. One study showed that full‐text downloads were almost twice as high for open access journals as opposed to journals with a paywall (https://openscience.com/open-access-increases-citation-a-brief-overview-of-two-reports/). However, the same article concluded that publishing an article in an open access journal does not necessarily influence the number of citations, not at least in the first 9–12 months.

An important consideration is that open access journal readers have access to your article independently of their libraries’ journal collection. This allows a much higher visibility and discoverability of your publication, as there are no restrictions in viewing it. Open access articles are available to everyone on a global scale, even to users in institutions that do not have access to a large number of subscription journals. This can significantly broaden the readership and usage of an article. I remember working as a clinical physicist at an academic institution with a medical library about a 10‐min walk from the cancer center. Even with my best intentions, it was difficult to carve out 2‐ to 4‐h time slots for library research. How much better to grab the article immediately and find the information I need without leaving my desk.

Respecting copyright, Wiley uses the Creative Commons Attribution 3.0 Unported (CC BY 3.0) license. Wiley publishes the following notice within its instructions to authors of JACMP articles:

## COPYRIGHT NOTICE

Authors who publish with *Journal of Applied Clinical Medical Physics* agree to the following terms:

- Authors retain copyright and grant the journal right of first publication with the work simultaneously licensed under a Creative Commons Attribution License that allows others to share the work with an acknowledgement of the work's authorship and initial publication in this journal.
- Authors are able to enter into separate, additional contractual arrangements for the non‐exclusive distribution of the journal's published version of the work (e.g., post it to an institutional repository or publish it in a book), with an acknowledgement of its initial publication in this journal.
- Authors are permitted and encouraged to post their work online (e.g., in institutional repositories or on their website) prior to and during the submission process, as it can lead to productive exchanges, as well as earlier and greater citation of published work. (See the Effect of Open Access). https://authorservices.wiley.com/open-science/open-access/for-authors/faqs.html

The impact of this license is that you retain copyright and reuse rights of your work. Your work can be shared, copied and redistributed in any medium or format under the condition that you as the author are given appropriate credit. A link to the license must be provided, and changes made have to be indicated. No one may use it commercially or alter it without your permission. Finally, publishing under this license means that you comply with open access mandates as may be required by a granting agency respecting your article. By retaining your rights, you will be permitted to:

- Maintain the right to disseminate your work
- Maintain the right to use your work for educational purposes including your classes
- Maintain the right to post your work on any website you choose, including your own website
- Maintain the right to post and archive your work in your employer's Institutional Repository
- Reserve the right to post the pre‐refereed or even post‐refereed version of your paper
- Allow for the largest possible audience

The bottom line is that by owning your article and retaining legal title to the ideas and data contained therein, you control how it is used. For clinical physicists who want to disseminate their work as widely and rapidly as possible to benefit our community and our patients, the JACMP is a good place to publish.

